# Prescription Department

**Published:** 1897-03

**Authors:** 


DEBILITY, GENERAL AND SENILE.

℞. Quiniæ sulphat........gr. xxx.

Acidi sulphurici dil....q. s. ad.

ft. sol..................

Aquæ.... ................℥ij.

Tinct. ferri chlor.........℥ss.

Spts. chloroformi..........ʒvj.

Glycerinæ.................℥iv.

M. Sig.: A teaspoonful three times daily.—*Loomis.*

CATARRH, NASAL AND FAUCIAL.

℞. Sodii salicylat.............ʒij.

Sodii biborat..............ʒiij.

Glycerinæ..................ʒiv.

Aquæ..... ........q. s. ad ℥vj.

M. Sig.: A desertspoonful in a pint of water, used with spray or douche.—*Bean.*

SCIATICA.

℞. Tinct. colchici sem.....gtt. xv.
Potassii iodidi..........gr. x.
Tinct. zingiberis.......gtt. x.
Syrupi simplicis........
Aquæ...........aa q. s. ad ℥ij.
M. Ft. haustus. Sig.: Apply a strip of blistering plaster over the course of the nerve, and give the above draught in water thrice daily between meals.—*Da Costa.*

FISTULÆ.

℞. Ext. sanguinariæ fld...... .℥ij.
Sig.: Inject a quantity sufficient to fill and distend the fistula.—*Phillips.*

